# Motion Constraints and Vanishing Point Aided Land Vehicle Navigation

**DOI:** 10.3390/mi9050249

**Published:** 2018-05-20

**Authors:** Zhenbo Liu, Naser El-Sheimy, Chunyang Yu, Yongyuan Qin

**Affiliations:** 1 School of Automation, Northwestern Polytechnical University, Xi’an 710129, China; qinyongyuan@nwpu.edu.cn; 2 Department of Geomatics Engineering, University of Calgary, Calgary, AB T2N 1N4, Canada; elsheimy@ucalgary.ca (N.E.-S.); chunyang.yu@ucalgary.ca (C.Y.)

**Keywords:** inertial navigation system, vanishing point, nonholonomic constraints, Extended Kalman filter, Stochastic Cloning Kalman filter, relative attitude, MEMS inertial measurement unit

## Abstract

In the typical Inertial Navigation System (INS)/ Global Navigation Satellite System (GNSS) setup for ground vehicle navigation, measures should be taken to maintain the performance when there are GNSS signal outages. Usually, aiding sensors are utilized to reduce the INS drift. A full motion constraint model is developed allowing the online calibration of INS frame with respect to (w.r.t) the motion frame. To obtain better heading and lateral positioning performance, we propose to use of vanishing point (VP) observations of parallel lane markings from a single forward-looking camera to aid the INS. In the VP module, the relative attitude of the camera w.r.t the road frame is derived from the VP coordinates. The state-space model is developed with augmented vertical attitude error state. Finally, the VP module is added to a modified motion constrains module in the Extended Kalman filter (EKF) framework. Simulations and real-world experiments have shown the validity of VP-based method and improved heading and cross-track position accuracy compared with the solution without VP. The proposed method can work jointly with conventional visual odometry to aid INS for better accuracy and robustness.

## 1. Introduction

Accurate vehicular navigation is of great importance for some core parts in “smart cities”. It is not only used in the Guidance, Navigation and Control systems for autonomous driving, but also in V2X (vehicle-to-everything) technologies for effective transportation and cooperative safety communications among vehicles. For example, the positioning and heading information is shared among the V2V (vehicle-to-vehicle) network, according to the Cooperative Awareness Message (CAM) and Basic Safety Message (BSM), from the European Telecommunications Standards Institute (ETSI) and US Society of Automotive Engineers (SAE), respectively [1,2]. The inertial navigation system (INS) has the sole capability to produce a complete and continuous set of navigation state data, with high precision during a short time span. However, the positioning error grows considerably with time, especially when using low-cost MEMS inertial measurement units (IMU). Therefore, INS should be integrated with other aiding sensors. INS and Global Navigation Satellite System (GNSS) integration is commonly used for outdoor vehicles navigation. Nevertheless, GNSS may not be available in tunnels, and can suffer from obstruction and multipath errors in city centers and mountainous regions. There is also a possibility of a GNSS receiver being jammed or spoofed [3,4].

Various aiding sensors can be used to mitigate the error growth of INS in GNSS denied environment, such as motion constraints (e.g., Non-holonomic Constraints (NHC) as a “virtual” sensor) [5], speed sensors (wheel odometers, Doppler radars) [6], LiDAR sensors [7], and digital maps for map-matching or map-aiding [8,9]. Recently, many researchers proposed to use vision sensors to aid the navigation system, in loosely coupled form, with visual odometry (VO) module [10,11] or in tightly coupled form [12,13,14], and achieved remarkable results. Apart from commonly used point features in the pose estimation, vanishing points extracted from parallel lines in the scene can be used to obtain attitude information, which in then fused with INS in a loosely coupled manner. A simple and fundamental work of determining the rotation between two camera views was introduced by [15]. Rotation matrix between the two camera coordinate systems can be solved linearly when one obtains coordinates of three non-collinear vanishing points represented in both images. An alternative method using only two dominant directions is presented by [16] based on the Rodrigues’ formula. The vanishing point-based attitude estimation method has been investigated to help indoor pedestrian navigation or improve VO performance in hallway environments [17,18,19] and UAV navigation in urban areas with structured buildings [20,21].

For land vehicle applications, Kim et al. [22] showed improved accuracy when adding the omnidirectional vision attitude module to the INS/odometer integration for car navigation with a “Manhattan world” assumption [23] during GPS outages. These methods rely on the observation of parallel lines in structured buildings. However, there may be no sufficient building observations, for example, when driving through dense treed areas. Parallel lane markings or boundaries can be generally observed and detected in the structured road. Previous research considered ground plane tracking [24], camera calibration [25], and vehicles’ in-lane lateral distance calculation or prior road map-based shape registration for better localization performance [26,27,28]. Basically, these methods use observed lane markings for relative lateral positioning. Few works have been done on the navigation aiding using the vanishing point of parallel lane markings. In the earlier work, we used VP measurements of parallel lane markings to aid the INS onboard a car [29]. The relative heading aiding is treated in an absolute way, i.e., using true heading aiding method, which may cause large positioning error after certain time during GNSS outages. As the heading error grows larger, the measurement will be less relevant to the true heading error.

For the estimator used for integrated navigation, EKF (Extended Kalman filter) is most widely used due to its computation efficiency and the fact that nonlinearities of the error system model and measurement model are moderate in common situations. There are other nonlinear filters that do not linearize the system model at all, such as the Unscented Kalman filters (UKF) and particle filters (PF) [30,31,32]. For the application of GPS/INS navigation system, a performance comparison between EKF and UKF was made in [31]. It is reported that EKF and UKF offer identical performance except for unrealistic situation, e.g., a sixty kilometres initial position error. For the in-motion alignment of a low-cost INS with large initial attitude errors, the UKF outperforms EKF [32]. Recently, a dual Kalman filter method [33,34] is developed to estimate input and state simultaneously, which is suitable for structures fatigue damage identification. However, in the inertial navigation-based multi-sensor integration, the inputs are from IMU sensor readings, which are already known. The sensor biases are usually augmented into the state vector.

In this paper, the motion constraints module with a full module and the VP module are developed to aid the INS in the framework of EKF. We develop the VP aiding method based on the idea of Stochastic Cloning Kalman filter [35,36] considering the relative nature of the attitude measurement, which is also the estimation tool in the application of Micro Aerial Vehicle indoor navigation [37]. Using the sequential updating EKF, the proposed method can be easily incorporated into the conventional VO-based loosely-coupled vision-aided inertial navigation system (VINS) to improve the accuracy and robustness.

The paper is organized as follows. The coordinate systems involved are defined and the relationship is established between VP 2D coordinates and relative attitude of the camera frame w.r.t. the road frame. Based on this relationship, the VP aiding module is developed with an augmented state. VP module is then added into existing motion constraint aided INS. We evaluate the proposed algorithm by the Monte Carlo simulations and real data experiments. Discussions and conclusions are presented in the end.

## 2. Relative Attitude from Vanishing Point Coordinates

### 2.1. Coordinate Systems Definition

**Figure 1:**
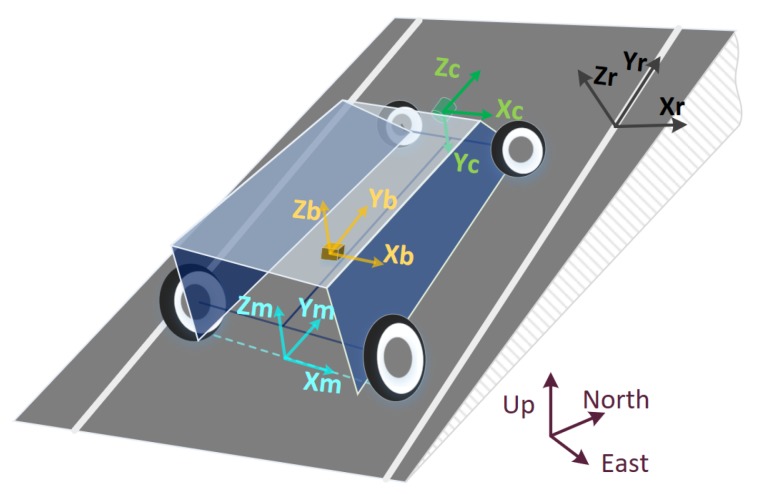
Coordinate systems.

The coordinate systems defined here follow the right hand rule as shown in Figure 1 [29].

1. Navigation frame (*n*-frame): For the near-Earth navigation, it is defined as the local-level frame where *x*, *y*, *z* axes are in the directions of east, north and up.
2. Vehicle motion frame (*m*-frame) [38]: *x*-axis is parallel with the non-steered axle, pointing to the right, *y*-axis points to the forward, and *z*-axis points up. Both *y* and *z*-axes are in the vertical plane of symmetry. The origin, which is the measurement origin of the vehicle, is the position at road height, mid-way between the wheels of non-steered axle.
3. IMU body frame (*b*-frame): *x*-axis points right, the *y*-axis points forwards and the *z*-axis points up. The origin is the measurement origin of the IMU.
4. Camera frame (*c*-frame): The camera is looking forward, so the *z*-axis points forward, *x*-axis points right, and *y*-axis points down.
5. Camera intermediate frame ($c^{\prime }$-frame): rotate 90${}^{\circ }$ about *x*-axis of camera frame to get camera intermediate frame. It is introduced to derive the relationship between the vanishing point coordinates and relative attitude of the camera.
6. Road markings frame (*r*-frame): fixed to a road and rotated with the road terrain and road direction. Suppose the vehicle is moving on the road with parallel lane markings. In *r*-frame, *x* and *y* axes are in the road plane, perpendicular to and along the lane markings, respectively. *z*-axis is perpendicular to and pointing out from the road surface.

### 2.2. Camera Relative Attitude Derived from Vanishing Point Coordinates

**Figure 2:**
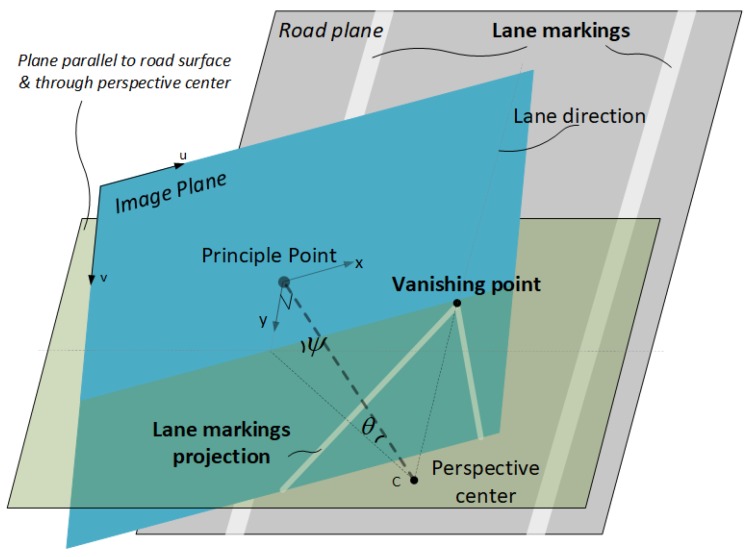
Vanishing point and relative attitude.

A vanishing point (VP) is the point of intersection of image projections of a set of parallel 3D lines, e.g., lane-lines. Each set of parallel lines is associated to a VP in an image. As shown in Figure 2, the VP of the parallel lines is the intersection of the image plane with a ray parallel to the world lines through the camera center. The VP image coordinates are not affected by the relative translation, but only affected by the relative rotation between the camera and the scene [39]. This property enables the VP-based module a tool to determine the camera-to-scene relative attitude, which can be utilized as an aiding source for the INS. It is known that at least two sets of parallel lines are needed to determine all three degree of freedom of a camera’s relative attitude [16].

Here we define some Euler angles and rotation matrices that are important for our proposed methods. The rotation matrix from the *r*-frame to the $c^{\prime }$-frame ($C_{c^{\prime }}^{r}$) can be represented by a set of Euler angles (θ,γ,ψ), which denote the relative pitch, relative roll and relative yaw, respectively. The rotation order is as follows. First rotate ψ about *z*-axis of *r*-frame, then θ about *x*-axis, and finally γ about *y*-axis. When the first two rotations are completed, as illustrated in Figure 2, we derive the image coordinates of vanishing point

(1) $$\begin{array}{c} x_{vp}^{1}=x_{c}+\frac{f}{\cos\theta }\tan\psi \\ y_{vp}^{1}=y_{c}+f\tan\theta \end{array}$$

where *f* is the focal length of the camera, $x_{c}$ and $y_{c}$ are the coordinates of principle point. After the 3rd rotation, the final vanishing point coordinates ($x_{vp},y_{vp}$) can be expressed as

(2) $$\left[\begin{array}{c} x_{vp}-x_{c}\\ y_{vp}-y_{c} \end{array}\right]=\left[\begin{array}{cc} \cos\gamma & \sin\gamma \\ -\sin\gamma & \cos\gamma \end{array}\right]\left[\begin{array}{c} x_{vp}^{1}-x_{c}\\ y_{vp}^{1}-y_{c} \end{array}\right]$$

Therefore, we have

(3) $$\begin{array}{c} x_{vp}=x_{c}+\frac{f}{\cos\theta }\tan\psi \cos\gamma +f\tan\theta \sin\gamma \end{array}$$

(4) $$\begin{array}{c} y_{vp}=y_{c}-\frac{f}{\cos\theta }\tan\psi \sin\gamma +f\tan\theta \cos\gamma \end{array}$$

From Equations (3) and (4), one can find that there are two knowns ($x_{vp}$ and $y_{vp}$), and three unknowns (θ,γ,ψ); therefore, additional constraints are needed to solve the equations.

Here we derive the relative yaw angle from the vanishing point coordinates. Combine Equations (3) and (4) and cancel out the relative yaw angle ψ, then we have

(5) $$\left(x_{vp}-x_{c}\right)\sin\gamma +\left(y_{vp}-y_{c}\right)\cos\gamma =f\tan\theta$$

From Equation (3), we can obtain relative yaw of *c*-frame w.r.t. *r*-frame.

(6) $$\psi =\arctan\frac{\left(\frac{x_{vp}-x_{c}}{f}-\tan\theta \sin\gamma \right)\cos\theta }{\cos\gamma }$$

Here we assume the relative roll is zero,

(7) $$\hat{\gamma }=0$$

Then the relative pitch and yaw can be expressed as

(8) $$\begin{array}{c} \hat{\theta }=\arctan\frac{y_{vp}-y_{c}}{f} \end{array}$$

(9) $$\begin{array}{c} \hat{\psi }=\arctan\left(\frac{x_{vp}-x_{c}}{f}\cos\hat{\theta }\right) \end{array}$$

Note that the relative attitude here is the camera intermediate frame $c^{\prime }$ w.r.t. the road frame.

### 2.3. Straight Lane Determination and Vanishing Point Detection

Lane marking detection has been widely researched in the literature of autonomous driving and is gradually being incorporated into vehicles navigation modules [40]. In this paper, the measurement is derived from the image observations of parallel straight lane markings, so straight lane detection should be performed before the VP extraction. The VP extraction is based on the commonly used Hough transform which can extract dominant lines in the binary edge image.

**Figure 3:**
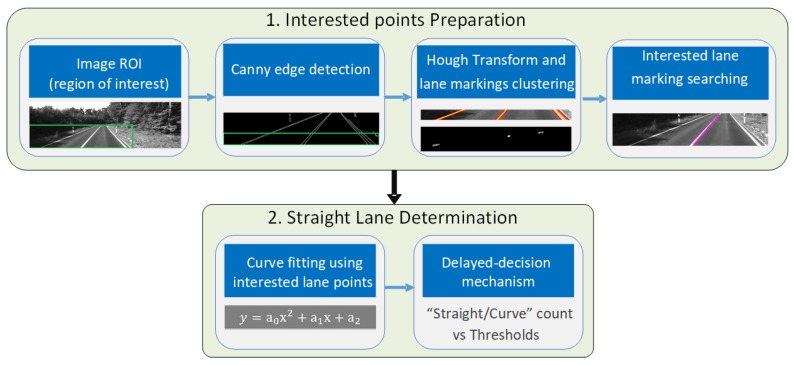
Straight Lane Detection Procedure.

The following will provide more details on the straight lane detection module (shown in Figure 3). First, the region of interest (ROI) is selected from the original image, and the edges are detected using the Canny Edge detector. The Hough transform is applied to the bottom ROI, which is near straight, to get multiple straight line parameters (polar coordinates ρ−θ space), which are grouped with into several lane marking groups using DBSCAN (Density-Based Spatial Clustering of Applications with Noise) clustering. Only one lane marking in the bottom ROI is selected, and we obtain the interested lane marking points by searching the binary image from bottom to top with small regions along the main direction trend. Then the straight lane marking detection consists of two parts: (1) curve-fitting using interested lane points to suggest initial decisions of straight or curved lane, and (2) a delayed-decision mechanism based on the accumulated initial decisions among frames.

For the first part, the interested lane marking points’ coordinates are stored for a quadratic polynomial curve fitting. The initial straight detection criterion is the comparison between estimated coefficient and the threshold of the second-degree term. Erroneous points may be selected as interested lane points, for example, the straight lane may be wrongly identified as a curved lane. For this reason, we add second part, a simple “delayed” decision mechanism to obtain final results. It works as follows: the initial decisions of both “straight” or “curve” lanes are accumulated among image frames. At least $N_{c}$ curve detections suggest a curved lane, and at least $N_{s}$ straight lane detection after a long-curved segment will determine a straight lane. The values of the thresholds ($N_{c}$ and $N_{s}$) in the decision mechanism are based on the velocity of the vehicle, heading change, and so on.

### 2.4. Measurement Uncertainty

Because the relative attitude measurement will be fused with INS solutions, it is essential to determine the measurement uncertainty. The error sources include IMU-camera calibration error, zero-roll assumption error, vanishing point detection error, and so on. We assume the accurate inter-sensor calibration, so calibration error is not considered here.

Now we investigate the measurement uncertainty caused by the zero-roll assumption error. As presented in the following subsection, relative yaw is the main aiding source, so we conduct a sensitivity analysis on the relative yaw w.r.t. the zero-roll assumption error.

From Equations (3), (4) and (6), we derive the partial derivation of ψ w.r.t. γ

(10) $$\frac{\partial \psi }{\partial \gamma }=\cos^{2}\psi \left(\tan\psi \tan\gamma -\sin\theta \right)$$

Assuming the zero-roll assumption error is ▵γ, the induced relative yaw error is

(11) $$▵\psi =\frac{\partial \psi }{\partial \gamma }▵\gamma =\cos^{2}\psi \left(\tan\psi \tan\gamma -\sin\theta \right)▵\gamma$$

**Figure 4:**
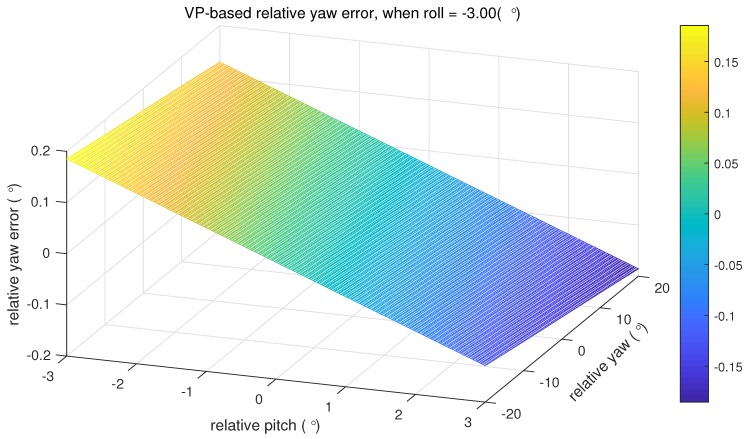
zero-roll assumption induced relative yaw error distribution.

We set an example for better illustration of how large the induced error can be. The ranges of the relative yaw and the relative pitch are set $\left[-20^{\circ },20^{\circ }\right]$ and $\left[-3^{\circ },3^{\circ }\right]$, respectively. The true relative roll is set $-3^{\circ }$ (which means the relative roll error is $-3^{\circ }$). As shown in Figure 4, the maximum absolute value of ▵ψ is less than $0.2^{\circ }$, occurring at the maximum relative pitch and yaw values.

For the error of VP detection, it usually comes from the Hough transform in the line detection. The empirical VP uncertainty is set as pixel level. The measurement errors can also come from the faulty identifying curved lane marks as straight, which should be avoided as much as possible. Due to the nature of inertial navigation, an incorrectly identified straight lane marking can lead to larger errors in the navigation solution than a straight line not found. Therefore, our strategy is to give the relatively strict thresholding as illustrated in the “Straight Lane Determination” procedure in Figure 3. The influence of false detections will be presented in the discussion section.

## 3. Sensor Fusion Process

This section describes the system model, measurement model and EKF used for the sensor integration using vehicle motion constraints, odometer information and vanishing point observations. In this paper, we make following assumptions of the system:

(1) The vehicle is moving on a certain road segment, where the road boundaries or lane markings are parallel;
(2) The camera has been calibrated, i.e., the Interior Orientation Parameters (IOPs) are known. The relative rotations between IMU frame and camera frame are also known.

### 3.1. Filter State

The navigation state is presented by the position (latitude *L*, longitude λ and height *h*), the velocity (east velocity, north velocity, and up velocity), and the attitude (pitch, roll, and heading). Differential equations of position $P^{n}$, velocity $V^{n}$, and attitude matrix $C_{b}^{n}$ are

(12) $${\dot{P}}^{n}=\left[\begin{array}{c} \dot{L}\\ \dot{\lambda }\\ \dot{h} \end{array}\right]=\left[\begin{array}{ccc} 0 & \frac{1}{R_{M}+h} & 0\\ \frac{1}{(R_{N}+h)\cos L} & 0 & 0\\ 0 & 0 & 1 \end{array}\right]V^{n}$$

(13) $${\dot{V}}^{n}=C_{b}^{n}f^{b}-\left(2\omega_{ie}^{n}+\omega_{en}^{n}\right)\times V^{n}+g^{n}$$

(14) $${\dot{C}}_{b}^{n}=C_{b}^{n}\left[\omega_{ib}^{b}\times \right]-\left[\left(\omega_{ie}^{n}+\omega_{en}^{n}\right)\times \right]C_{b}^{n}$$

where $C_{b}^{n}$ is the attitude matrix, $R_{M}$ and $R_{N}$ are meridian radius of curvature and prime vertical radius respectively, $f^{b}$ is the specific force vector measured by the accelerometers, and $\omega_{ib}^{b}$ is the angular rate vector of the body frame w.r.t. the inertial frame, measured by the gyroscopes. $\omega_{ie}^{n}$ is the earth rotation angular rate vector w.r.t. the inertial frame, and $\omega_{en}^{n}$ is the angular rate of the navigation frame w.r.t. ECEF frame. $g^{n}={\left[0\;\;0\;-g\right]}^{T}$ is the gravity acceleration vector in navigation frame, and *g* is the magnitude of local gravity acceleration. The notation [a×] is the asymmetric matrix of a vector a. INS mechanization is the process to calculate the navigation states, propagated through Equations (12)–(14) by using numerical integration methods, given the initial navigation states and IMU measurements [41,42].

The navigation states can be considered as the full states, and the associated errors are called error states. For the EKF-based integrated navigation systems, error states are estimated and used to correct the full states once the measurement update is accomplished [43]. For convenience, the filter state refers to the error state, which is denoted as X. For the normal INS-based EKF, the state is a 15-dimensional vector, not including the scale factors of the IMU sensor:

(15) $$X_{I}={\left[\begin{array}{ccccc} \delta V^{nT} & \phi^{nT} & \delta p^{T} & b_{g}^{T} & b_{a}^{T} \end{array}\right]}^{T}$$

where the navigation state error vectors $\delta V^{n}$, $\phi^{n}$, and δp are the velocity errors, attitude errors, and position errors, respectively. INS error model describes the differential equations of navigation state errors. $b_{g}$ and $b_{a}$ are the bias vectors of gyroscopes and accelerometers, each element of which is modeled as a 1st order Gauss-Markov process.

Here we augment two groups of states, one for the motion constraints aiding and the other for the VP aiding.

(16) $$X={\left[\begin{array}{ccc} X_{I}^{T} & \underset{\mathrm{motion}\;\mathrm{constraint}}{\underset{︸}{\begin{array}{cccc} \delta \beta_{x} & \delta \beta_{z} & K_{od} & \delta r^{bT} \end{array}}} & \underset{\mathrm{VP}-\mathrm{aiding}}{\underset{︸}{\phi_{U}^{0}}} \end{array}\right]}^{T}$$

where $\delta \beta_{x}$ and $\delta \beta_{z}$ are the misalignment errors between *b*-frame and *m*-frame about *x* and *z* axes. $\delta r^{b}$ is the lever-arm error vector of the IMU measurement origin w.r.t. the origin of *m*-frame. $K_{od}$ is the scale factor error of the odometer. $\phi_{U}^{0}$ is the cloned state of the third component of $\phi^{n}$ at the beginning of the detection of a straight lane segment. Thus, the dimension of the system state is 22.

The reason we augment the error state of $\delta \beta_{x}$, $\delta \beta_{z}$, $K_{od}$ and $\delta r^{b}$ is to achieve accuracy improvement of the system. Specifically, these values may not be calibrated beforehand or may not be accurate. Also, values can change in various driving scenarios due to the suspending system and different tyre pressures. In this paper, $\delta \beta_{x}$, $\delta \beta_{z}$, $K_{od}$ and $\delta r^{b}$ are modeled as random constants. $\phi_{U}^{0}$ is also modeled as a random constant because it will not propagate with time.

### 3.2. Motion Constraint Aiding

Here Non-holonomic Constraint (NHC) is applied as the velocity constraint along the body *x* and *z* axes. It is based on the assumption that the land vehicle does not jump off the ground or slide sideways under normal conditions. The forward velocity can be derived from the wheel odometer if available. In this condition, the odometer measurement and pseudo-measurement of lateral and vertical velocity in the vehicle motion frame are formed as

(17) $${\tilde{V}}_{\mathrm{veh}}^{m}=\left[\begin{array}{c} 0\\ {\tilde{V}}_{od}\\ 0 \end{array}\right]=\left[\begin{array}{ccc} 1 & 0 & 0\\ 0 & 1+K_{od} & 0\\ 0 & 0 & 1 \end{array}\right]V_{\mathrm{veh}}^{m}+v_{V}$$

where $V_{\mathrm{veh}}^{m}$ is the vehicle velocity in *m*-frame, ${\tilde{V}}_{\mathrm{veh}}^{m}$ is the measurement of NHC and the velocity indicator, and $v_{V}$ is the measurement noise vector.

Based on the equation of relative linear motion in [44], a basic relationship exists between IMU body frame velocity and vehicle frame velocity:

(18) $$V_{\mathrm{veh}}^{m}=C_{b}^{m}\left(V^{b}+\left[\omega_{eb}^{b}\times \right]r^{b}\right)$$

where $V^{b}$ is the IMU velocity in the b-frame, $\left[\omega_{eb}^{b}\times \right]$ is the cross product of the angular velocity of the *b*-frame w.r.t. Earth-centered Earth-fixed (ECEF) frame, $r^{b}$ and $C_{b}^{m}$ are the lever-arm vector and the rotation matrix between the *m*-frame and the *b*-frame.

Based on Equation (18), we can estimate ${\hat{V}}_{\mathrm{veh}}^{m}$ from INS as follows,

(19) $${\hat{V}}_{\mathrm{veh}}^{m}={\hat{C}}_{b}^{m}\left({\hat{C}}_{n}^{b}{\hat{V}}^{n}+\left[{\hat{\omega }}_{eb}^{b}\times \right]{\hat{r}}^{b}\right)$$

where ${\hat{V}}^{n}$ and ${\hat{C}}_{n}^{b}$ are the INS calculated velocity and attitude matrix. ${\hat{C}}_{b}^{m}$ and ${\hat{r}}^{b}$ are estimated from the calibration, otherwise through an initial guess (for example, an identity matrix and a zero vector, respectively, for nearly aligned *b* and *m* frames).

The residual measurement is formed by subtracting the INS derived vehicle and velocity from odometer and NHC pseudo-measurement.

(20) $$Z_{NHC/OD}={\hat{V}}_{\mathrm{veh}}^{m}-{\tilde{V}}_{\mathrm{veh}}^{m}$$

Neglecting the second-order error items, the measurement equation can be written as [29]

(21) $$Z_{NHC/OD}={\hat{C}}_{n}^{m}\delta V^{n}-{\hat{C}}_{n}^{m}\left[{\hat{V}}^{n}\times \right]\phi^{n}+{\hat{C}}_{b}^{m}\left({\hat{\omega }}_{eb}^{b}\times \right)\delta r^{b}+\left[\begin{array}{ccc} 0 & -V_{y} & 0\\ 0 & 0 & -V_{y}\\ V_{y} & 0 & 0 \end{array}\right]\left[\begin{array}{c} \delta \beta_{x}\\ \delta \beta_{z}\\ K_{od} \end{array}\right]+v_{V}$$

where $V_{y}$ is the forward speed measured by the speed sensor or calculated from INS.

NHC aiding acts as a virtual sensor for land vehicle navigation, and can be applied in most driving conditions. If there is no odometer in the system, we select the first and third rows of Equation (21) to formulate the measurement.

### 3.3. Vanishing Point Aiding

In Section 2, we calculated the relative attitude from vanishing point coordinates. Now we will describe how this information can be used to aid the INS. As usual, we construct the measurement to associate with the state.

When the VP is detected for the first time (at time $t_{0}$) on the straight lane segment, we can calculate the attitude of this road segment (${\hat{C}}_{r}^{n}$) w.r.t. the navigation frame:

(22) $${\hat{C}}_{r}^{n}={\hat{C}}_{b,0}^{n}{\tilde{C}}_{r}^{b,0}={\hat{C}}_{b,0}^{n}C_{c}^{b}{\left({\tilde{C}}_{c,0}^{r}\right)}^{T}$$

where ${\hat{C}}_{b,0}^{n}$ is the INS attitude matrix at $t_{0}$, $C_{c}^{b}$ is the IMU-camera rotation matrix, ${\tilde{C}}_{c,0}^{r}$ is the camera attitude matrix w.r.t the road frame at time $t_{0}$, which is calculated by

(23) $${\tilde{C}}_{c,0}^{r}={\tilde{C}}_{c^{\prime },0}^{r}C_{c}^{c^{\prime }}=f_{DCM}\left({\left[\hat{\theta },\hat{\gamma },\hat{\psi }\right]}_{t_{0}}\right)\cdot f_{DCM}\left([-90^{\circ },0,0]\right)$$

where $f_{DCM}\left(\cdot \right)$ is the function to calculate the direct cosine matrix from the Euler angles. The value of ${\tilde{C}}_{c,0}^{r}$ is then stored for later use. As the vehicle moves on this road segment (at time *t*), we obtain the relative attitude from the VP measurement.

(24) $${\tilde{C}}_{c,t}^{r}=f_{DCM}\left({\left[\hat{\theta },\hat{\gamma },\hat{\psi }\right]}_{t}\right)\cdot f_{DCM}\left([-90^{\circ },0,0]\right)$$

Then we can derive the attitude matrix ${\tilde{C}}_{b_{VP}}^{n}$ from VP module at time *t*

(25) $${\tilde{C}}_{b_{VP}}^{n}={\hat{C}}_{r}^{n}{\tilde{C}}_{c,t}^{r}C_{b}^{c}$$

The measurement matrix is constructed as:

(26) $$Z_{M}={\hat{C}}_{b}^{n}{\left({\tilde{C}}_{b_{VP}}^{n}\right)}^{T}$$

where ${\hat{C}}_{b}^{n}$ is the current attitude matrix from INS.

Then we derive the relationship between the measurement matrix and the state with small error angle assumption.

(27) $$Z_{M}\approx \left(I-\phi^{n}\times \right)C_{b}^{n}C_{r}^{b}\left(I+\vartheta_{VP,t}^{r}\times \right)\left(I-\vartheta_{VP,0}^{r}\times \right)C_{b,0}^{r}C_{n}^{b,0}\left(I+\phi_{0}^{n}\times \right)$$

Neglecting the second-order error items,

(28) $$Z_{M}=I-\left[(\phi^{n}-\phi_{0}^{n}+\vartheta_{VP,0}^{r}-\vartheta_{VP,t}^{r})\times \right]$$

where I is the 3-dimensional identity matrix, $\phi_{0}^{n}$ is the attitude error at time $t_{0}$, $\vartheta_{VP,t}^{r}$ and $\vartheta_{VP,0}^{r}$ are the relative attitude error from VP at current time instant *t* and time $t_{0}$, respectively. The vector form of the measurement is

(29) $$Z_{V}=-\frac{1}{2}\left[\begin{array}{c} Z_{M}\left(3,2\right)-Z_{M}\left(2,3\right)\\ Z_{M}\left(1,3\right)-Z_{M}\left(3,1\right)\\ Z_{M}\left(2,1\right)-Z_{M}\left(1,2\right) \end{array}\right]=\phi^{n}-\phi_{0}^{n}+\vartheta_{VP,0}^{r}-\vartheta_{VP,t}^{r}$$

Here we do not want to incorporate the erroneous pitch/roll information from VP, which may deteriorate the pitch/roll estimation. Hence, the final measurement is the third component of $Z_{V}$.

(30) $$Z_{VP}=\left[\begin{array}{ccc} 0 & 0 & 1 \end{array}\right]Z_{V}=\phi_{U}-\phi_{U}^{0}+v_{VP}$$

where $v_{VP}$ is the measurement noise.

### 3.4. State Augmentation Treatment

We treat the state $\phi_{U}^{0}$ as an augmented state, which is the cloned state of $\phi_{U}$ at the very beginning when a straight lane segment is detected. It is based on the idea of Stochastic Cloning Kalman filter [35,36]. Stochastic Cloning deals with the relative state measurement which depends on the current state as well as the previous state of the system. The core of Stochastic Cloning is considering the interdependencies (cross-correlation terms) between the previous states and current states. At the start time ($t_{0}$) of the relative measurement, the relevant state is cloned and augmented in the state vector, and the state covariance is also augmented with considering the cross-correlation between the original state and cloned state. This essential step is often called “re-initialization”, because there is a life-time for the cloned state. During the life-time of the cloned state, EKF will evolve the state covariance matrix properly as long as the cloned state is modeled as a random constant.

Denote the first 21 states as normal states $X_{n}$, and last cloned state as $X_{c}$, such that the state in Equation (16) is written as

(31) $$X={\left[X_{n}^{T}\;\;\;X_{c}^{T}\right]}^{T}$$

Once the start of a new straight line segment is detected, we re-initialize the cloned state augmentation. The re-initialization is established as follows:

1. Clone the value of $X_{n}\left(6\right)$ to $X_{c}$;
2. Set the state covariance as
  (32) $$P_{k}=\left[\begin{array}{c} I_{21}\\ J_{1\times 21} \end{array}\right]P^{nn}{\left[\begin{array}{c} I_{21}\\ J_{1\times 21} \end{array}\right]}^{T}=\left[\begin{array}{cc} P^{nn} & P^{nc}\\ P^{ncT} & P^{cc} \end{array}\right]$$

where $P^{nn}$ is the covariance matrix of normal state $X_{n}$, $P^{cc}$ is the covariance matrix of the cloned state $X_{c}$, and $P^{nc}$ is the cross-correlation between normal state and cloned state. $J_{1\times 21}$ is the Jacobian of $X_{c}$ to $X_{n}$, which is presented as

(33) $$J_{1\times 21}=\left[\begin{array}{cc} O_{1\times 3} & \left[\begin{array}{ccc} 0 & 0 & 1 \end{array}\right]O_{1\times 15} \end{array}\right]$$

From the calculation, we can find that $P^{cc}$ is the element of $P^{nn}$ at index of (6, 6), which is the variance of the attitude error about the up direction.

**Figure 5:**
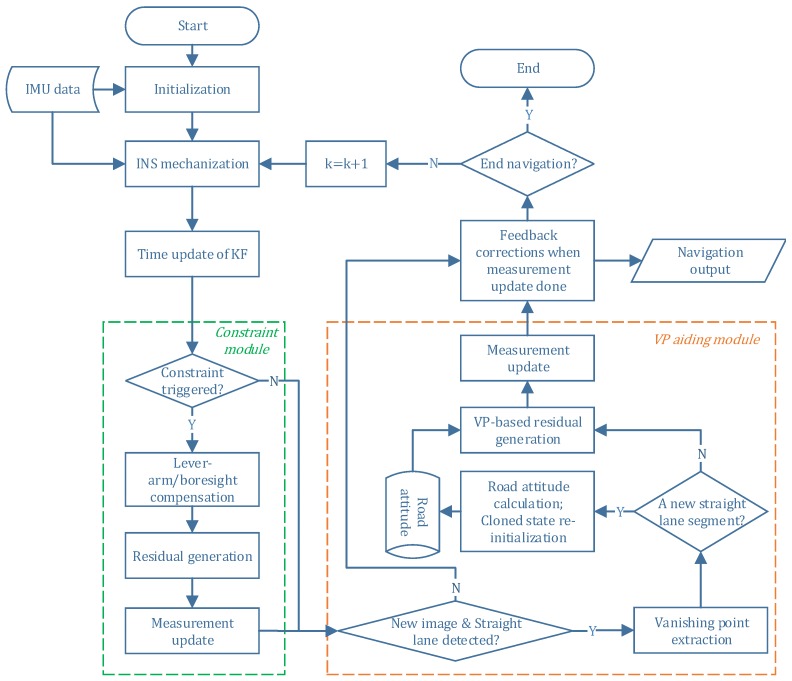
Flowchart of vanishing point and motion constraint aided INS.

The flowchart of integrating vanishing point module and motion constraint module with INS is illustrated in Figure 5. In the VP aiding module, when a set of new parallel straight lane markings is first detected, the road attitude of this segment is calculated and stored, which is used for future vehicle heading computation. Also, the state and covariance re-initiation is performed. Then subsequent VP observations can be used to construct the measurement residual information for measurement update.

## 4. Results

The described algorithm in the preceding subsections has been evaluated using simulations and real experiments. The results are given in this subsection. The simulation and experiments have shown the validity of using VP of lane markings to mitigate the INS heading error in order to achieve better positioning accuracy.

### 4.1. Simulation Results

**Figure 6:**
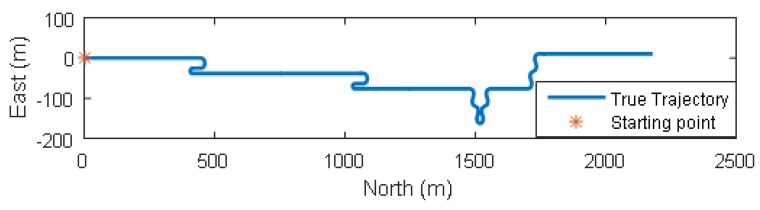
Horizontal trajectory.

**Figure 7:**
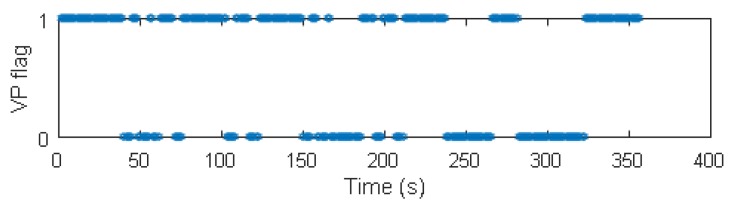
Valid vanishing point flags in the simulation.

We conducted Monte-Carlo simulations to evaluate the performance of VP aided land vehicle navigation algorithm. Specifically, we compared two schemes: INS/NHC/Odometer integration with and without VP aiding. A trajectory lasting 356 s was designed and generated. Several straight lines, turns, accelerations and climbing maneuvers were conducted. As shown in Figure 6, the main trend is along the north direction. The IMU, camera, odometer, GNSS parameters, lever-arm and boresight error of IMU are listed in Table 1. The distance traveled is 1571 m during the GNSS outage, which starts from 80 s till the end of the simulation. The valid VP flags over time are shown in Figure 7, indicating when the VP is used as a measurement.

**Figure 8:**
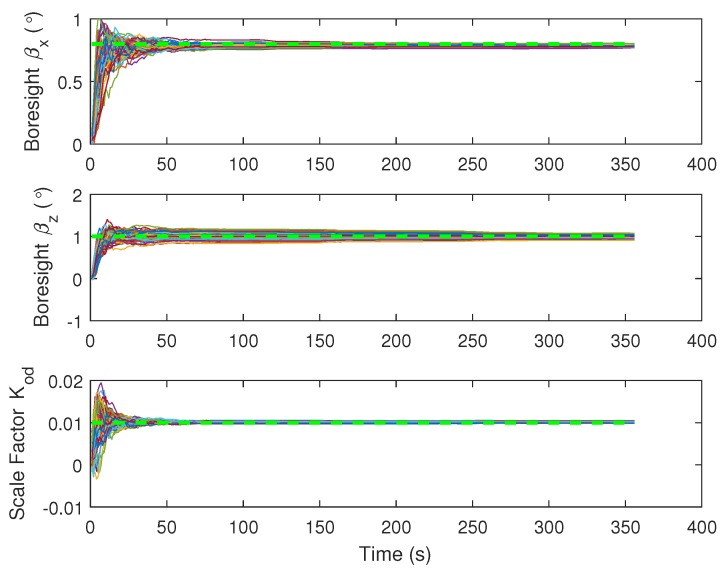
Boresight misalignment and scale factor of odometer (50 Monte Carlo trails estimation using INS/NHC/OD/VP are in thin solid lines; reference values are in thick dash green lines).

The estimation of IMU-vehicle frame boresight misalignment and scale factor of the odometer are shown in Figure 8. It can be seen that the boresight alignment angles $\beta_{x}$ and $\beta_{z}$ and the scale factor of the odometer $K_{od}$ can be quickly estimated in the first tens of seconds. The reason is that the vehicle undergoes some accelerations and decelerations during this period, and these maneuvers make the misalignment and scale factor observable [38]. The subsequent turns can also benefit their observability, contributing to good estimation accuracy.

**Figure 9:**
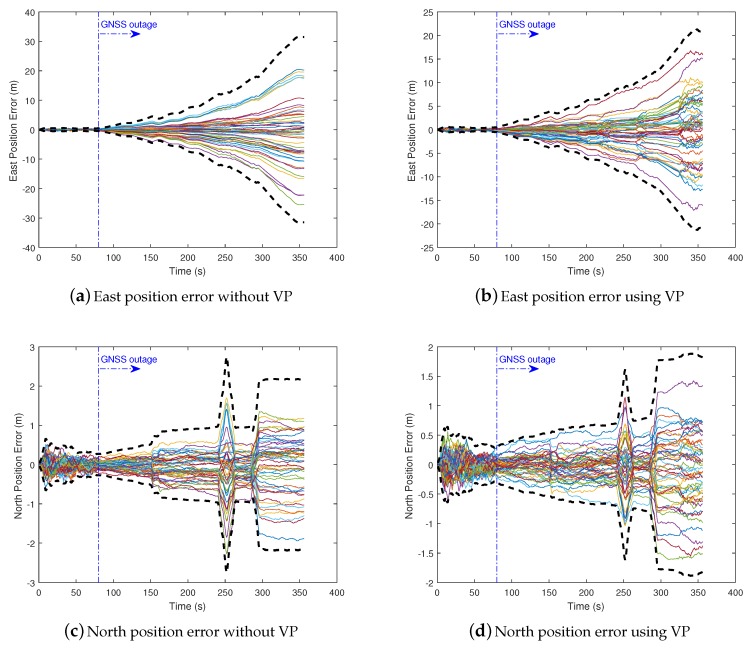
Position errors (50 Monte Carlo trials in solid lines and 3-sigma slope in dashed lines).

**Figure 10:**
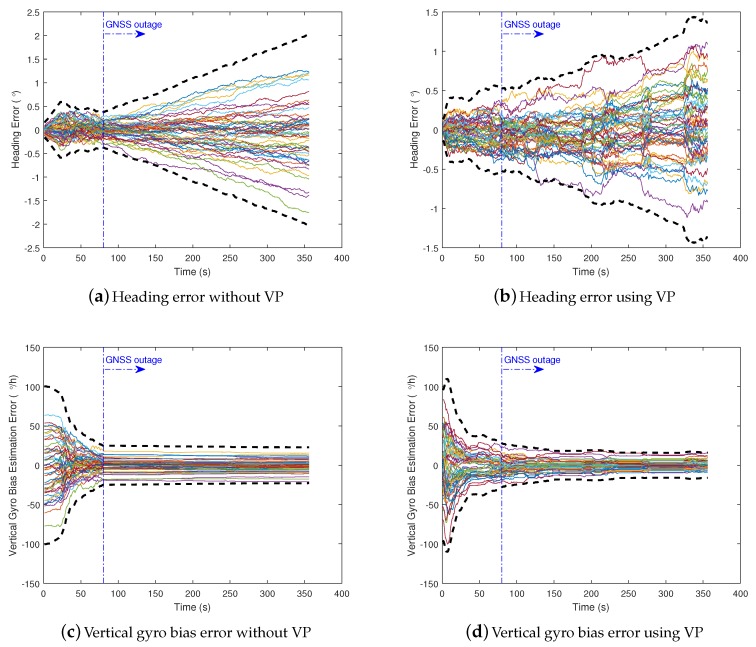
Heading and vertical gyro errors (50 Monte Carlo trials in solid lines and 3-sigma slope in dashed lines).

The east and north position error, heading error, and vertical gyro bias estimation error of two schemes with 50 Monte Carlo trials and 3-sigma slope are illustrated in Figure 9 and Figure 10. We can see from the 3-sigma RMS error slope that the overall performance of INS/NHC/Odometer with VP aiding method is better than the one without VP aiding. For the final cross-track error (east position error in this trajectory), the VP-based method achieves 0.32% DT (1 σ) with 33% improvement compared with no VP method ( 0.48% DT, 1 σ). The along track errors (north direction) are almost the same, and relatively small, for both schemes, because the forward velocity information from the odometer is utilized, which can benefit the forward positioning accuracy. As illustrated in Figure 10a,b, VP-based method has better heading accuracy, and the heading error growth is slower when the VP measurement is valid during GNSS outage. This is the primary reason for the improved performance of lateral positioning. For the vertical gyroscope bias estimation, we can see that when VP-aiding is used, nearly half of the bias has been estimated in the end, which outperforms the method without VP aiding (Figure 10c,d).

### 4.2. Experimental Results

The vanishing point and NHC aided fusion algorithm described in the preceding section were tested using “2011_09_26_drive_0028” and “2011_09_26_drive_0101” dataset (shortened as Path-0028 and Path-0101, respectively) from the KITTI benchmark dataset [45]. The raw IMU data (100 Hz) and rectified grayscale images (10Hz, global shutter) are used to verify the algorithm. The biases of gyroscopes and accelerometers are 36${}^{\circ }$/h and 1 mg, respectively. The reference is navigation results from an IMU/GPS data with the L1/L2 RTK positioning accuracy of 0.01 m, pitch/roll accuracy of 0.03${}^{\circ }$, and heading accuracy of 0.1${}^{\circ }$ [46]. A rough value of the lever-arm between IMU and the vehicle frame can be calculated based on the IMU’s mounted position. The boresight angles between IMU and the vehicle frame is unknown, so the initial values are assumed to be zeros.

To illustrate the performance of the proposed algorithms, we simulate the GPS outages on the trajectory. There is no wheel odometer or Doppler radar data in the dataset. For Path-0028, we compared the performance of the following navigation schemes.

(1) Free INS: Only INS mechanization is performed to calculate the navigation states.
(2) INS/NHC: NHC is triggered every 1 s with the measurement noise being 0.1 m/s (1 σ).
(3) INS/NHC/VP: Vanishing point aiding module is added into the system. It is triggered by the straight lane markings detection and valid vanishing point measurement.

For Path-0101, we added an odometer output module derived from reference data to perform the INS/NHC/OD integration. The performances are compared among free INS, INS/NHC/OD, and INS/NHC/OD/VP.

**Figure 11:**
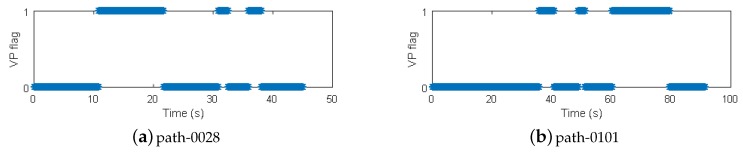
Valid vanishing point flags in the experiments.

In the VP detection, we select three Hough peaks in the image and get three intersections of three lines to have better accuracy and robustness. The vanishing point coordinates are determined by averaging the three intersections. A thresholding testing is conducted to detect the wrong lane marking selection. A $\chi^{2}$ statistic for the measurement residuals is generated for blunder detection to further improve the robustness of VP-based measurement update. The valid VP flags over time are recorded and shown in Figure 11 for Path-0028 and Path-0101, indicating when the VP can be used as a measurement.

#### 4.2.1. Results of experiment #1: Path-0028

**Figure 12:**
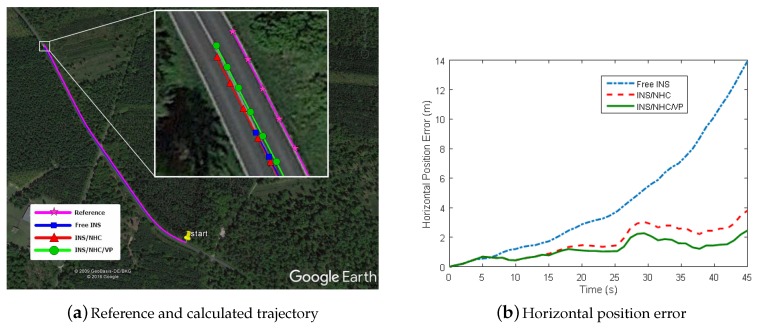
Position estimation results (path-0028).

The path-0028 is approximately 776m through dense trees and lasts 45 s (430 images), containing several direction changes of the path, as shown on Figure 12a. Here the whole trajectory of the GPS data was blocked except for the initial value of navigation states.

**Figure 13:**
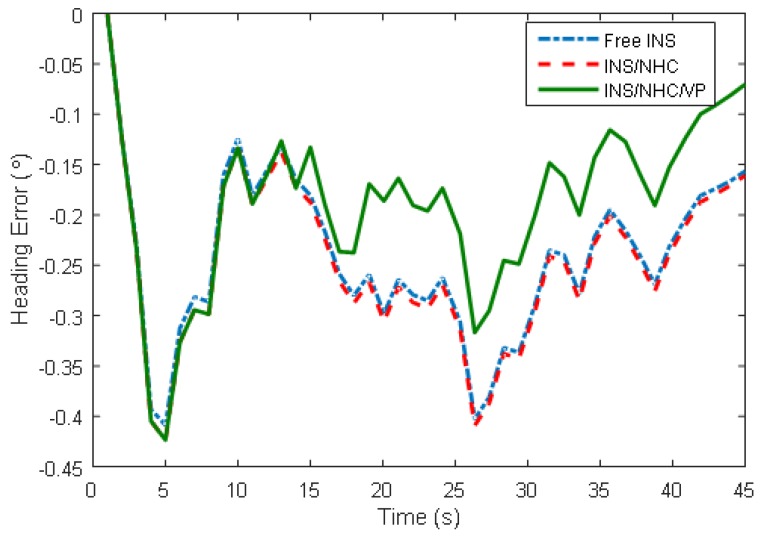
Attitude estimation error.

The position estimation results of the three navigation schemes are shown in Figure 12, and a summary of the navigation errors is given in Table 2. We can see that NHC/INS integration improves the positioning accuracy about 71% (horizontal error dropped to 4 m from 14 m). When the VP module is added, a further 33% improvement over INS/NHC scheme is achieved and the positioning error reduces to 0.32% DT. Pitch estimation gets improved when NHC is applied, while roll estimation does not. The reason is that there is a relationship between vertical velocity and pitch angle inherently in NHC, which makes the pitch angle observable. There is no improvement for pitch/roll estimation when VP is added on INS/NHC, since only relative yaw information is utilized. As illustrated in Figure 13, the heading error gets smaller when the VP-aiding works, which is the main reason for increased positioning accuracy. The vertical gyroscope bias (not shown here) is estimated to a value of 3${}^{\circ }$/h when using VP. In this sense, we can see the complementary benefits to the INS when using motion constraint and VP observation.

**Figure 14:**
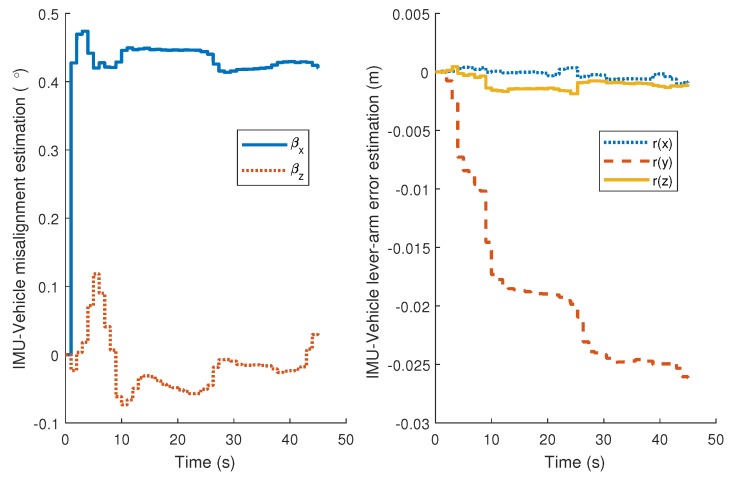
IMU-Vehicle relative pose estimation.

The estimation of relative pose (lever-arm and boresight misalignment) error between IMU frame and the vehicle frame is presented in Figure 14. The value of misalignment about the pitch axis reaches to about 0.4${}^{\circ }$. If the misalignment has not been augmented into the states, the induced velocity error in vertical direction will be 0.126 m/s under the speed of 18 m/s. A method to account for this is to increase the measurement noise variance of NHC. However, the biased measurement error will still degrade the system performance. State augmenting has the potential strength to achieve improved results under the condition that the augmented states are observable.

#### 4.2.2. Results of Experiment #2: Path-0101

**Figure 15:**
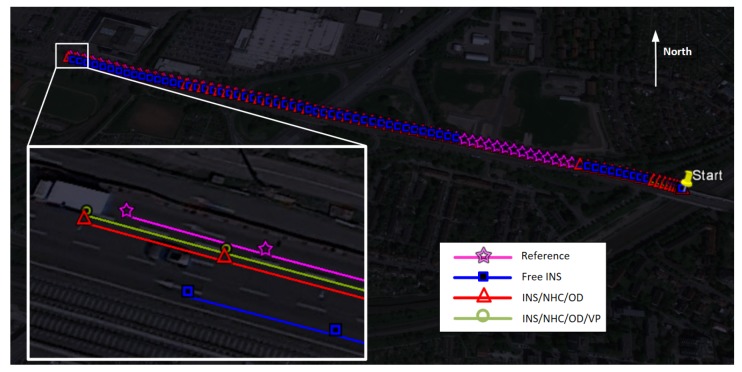
Reference and calculated trajectory on Google Earth (path-0101).

The path-0101 lasts 91 s, and the total traveled distance is about 1236 m with a standstill at the beginning. The simulated GNSS outage starts from 32 s until the end of trajectory. The positioning results and navigation errors are shown in Figure 15 and in Table 3, respectively. In the scheme of INS/NHC/OD, the horizontal position performance in the last epoch achieved 61% improvement compared with free INS. With the help of VP module, the cross-track error (most in north direction) is further improved over 50% to 0.153 m of RMS, and the heading RMS error is reduced to 0.167${}^{\circ }$ from 0.21${}^{\circ }$.

The lane markings in the whole trajectory are generally straight with several small curved segments. In the experiments, we find that if these small curved lane markings are also considered as straight and VP measurements are used, there will large variations in the heading and lateral position estimation, while the mean value of their errors are small. That is to say, small change of the lane marking direction will cause the heading estimation drift to the opposite direction, if the lane direction variation has not been detected. Thus, careful and strict treatment should be conducted on the straight lane detection module. We will discuss it in the following section.

## 5. Discussion

### 5.1. Robustness of VP-Aiding Approach

**Figure 16:**
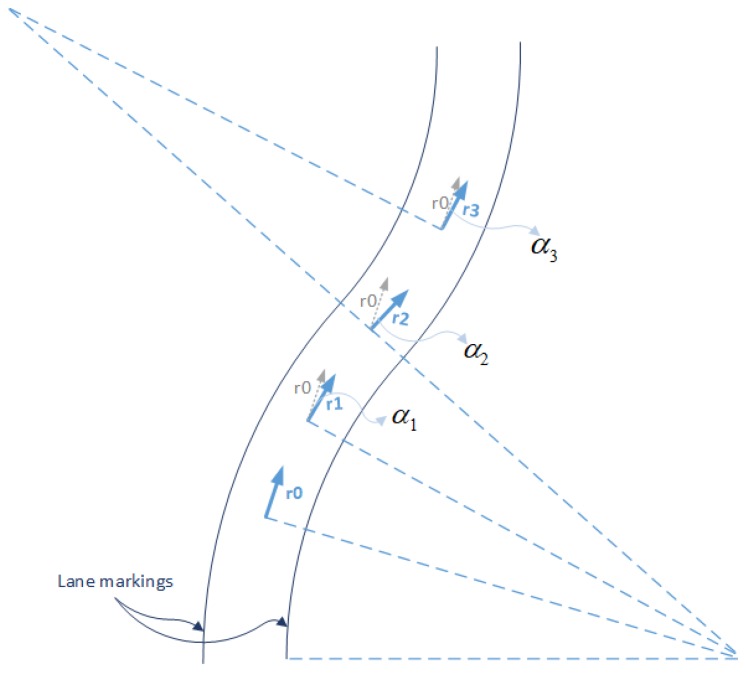
Curved lane markings and instant road frames.

There may still be the circumstances that the curvature of the lane mark is small, but wrongly identified as the straight line. Assume the vehicle moves in a curved road, while the camera detects the lane markings as straight. When the initial road frame $r_{0}$ is obtained, it will be registered and act as the reference frame when the camera is still observing the “same” segment. However, the true road frame will change along the curve road as shown in Figure 16, from $r_{1}$-frame to $r_{3}$-frame, for instance. Now we will describe its influence on the Kalman filter. In this situation, the attitude matrix from VP module described in Equation (25) will be changed to the following form.

(34) $${\tilde{C}}_{b_{VP}}^{n}={\hat{C}}_{r_{0}}^{n}{\tilde{C}}_{c,t}^{r_{i}}C_{b}^{c}$$

where the subscript $r_{0}$ is the initial road frame and the superscript $r_{i}$ (i=1,2,…) is the instant road frame along the road. After the derivation, the measurement in Equation (30) can be written as

(35) $$Z_{VP}=\left[\begin{array}{ccc} 0 & 0 & 1 \end{array}\right]Z_{V}=\phi_{U}-\phi_{U}^{0}-\alpha_{i}+v_{VP}$$

where $\alpha_{i}$ is the angle from $r_{0}$-frame to $r_{i}$-frame around the vertical axis. The term $-\alpha_{i}+v_{VP}$ will be considered as the measurement noise. The sensitivity analysis turns out to be the analysis of measurement error $\alpha_{i}$ to the filter performance. The most desirable situation is that the road lane markings are randomly curved, i.e., the direction changes slightly from one side to the other randomly, such that $\alpha_{i}$ is considered to be zero-mean white noise. The worst case is that the road is bending to one direction, causing $\alpha_{i}$ continuously growing, which will violate the Kalman filter assumption. The heading error state will be erroneously estimated and the gyro bias in *z*-axis will also get influenced. This kind of “soft” failure can be detected, for example by using the AIME (Autonomous Integrity Monitored Extrapolation) method. The concept is to form the averaged normalized innovation from last *N* measurements [4]. The AIME innovation test statistic for the filter at time *k* is [47]

(36) $$s_{k}^{2}=\delta z_{\mu }^{T}C_{\mu }^{-1}\delta z_{\mu }$$

where

(37) $$C_{\mu }^{-1}=\sum_{i=k+1-N}^{k}C_{i}^{-1}$$

(38) $$\delta z_{\mu }=C_{\mu }\sum_{i=k+1-N}^{k}C_{i}^{-1}\delta z_{i}$$

where $\delta z_{i}$ and $C_{i}$ are filter innovation and corresponding covariance at time *i*. The test statistic has a chi-square distribution with the degree of freedom being the measurement vector size. The chi-square test is conducted for the fault detection. Here the test statistic $s_{k}^{2}$ has a chi-square distribution with one degree of freedom, since the VP measurement has one dimension. Two parameters should be set for the fault detection. One is the threshold $T_{\mathrm{AIME}}$ to be compared with the test statistic $s_{k}^{2}$. The other is the sample size *N*.

If $s_{k}^{2}$ exceeds $T_{\mathrm{AIME}}$, then the soft-fault is reported. The threshold $T_{\mathrm{AIME}}$ can be calculated or looked up in a table when the significance level (α) or confidence level (1−α) is given. Here we choose the normal significance level α=0.05 (false alarm rate), so that $T_{\mathrm{AIME}}=3.84$ with one degree of freedom. The selection of $T_{\mathrm{AIME}}$ is a trade-off between the detection sensitivity and false-alarm rate.

The selection of sample size *N* is a trade-off between detection sensitivity and response time. Larger the sample size is, easier the detection will be. However, it is also demanded to have faster response time for less contaminated state estimates. In our setup, *N* is selected as 50 for 10 Hz image rate, which means a 5-s time window for the fault detection. As shown in Table 4, it can also detect the very slowly drifting case (#4: radius = 8000 m) with 5 s time window. Larger drift can be detected more quickly, if multiple test statistics with different sample sizes are computed.

To verify the robustness of proposed algorithm, we simulate several trajectories, each of which consists of a straight lane and a curved lane with a different radius, the worst case mentioned above. The straight and curved parts both last 120 s, with the constant speed of 10 m/s.

The sensors’ specifications are listed in Table 1. The GNSS is available during first 80 s, while motion constraints are used throughout the trajectory. To have a better idea of how the curviness can affect the proposed method, we examine three VP-aiding schemes, namely,

(1) No VP: only motion constraints are used to aid the INS;
(2) VP: Proposed VP-aiding method is used without soft faults detection;
(3) VP & AIME: Proposed VP-aiding method is used and soft faults detection (AIME) is utilized.

**Figure 17:**
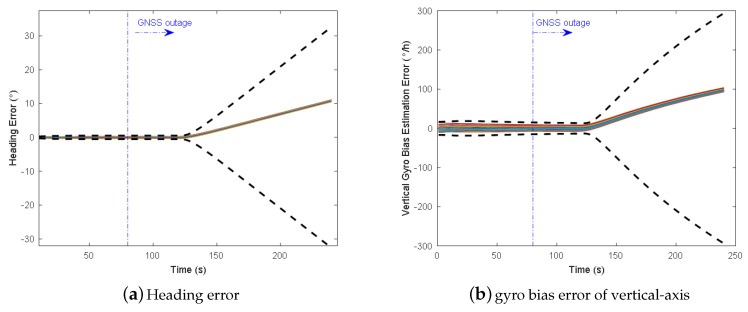
Heading and vertical-axis gyro bias estimation errors for trajectory No.3 without AIME (50 trials and 3-sigma error bound).

**Figure 18:**
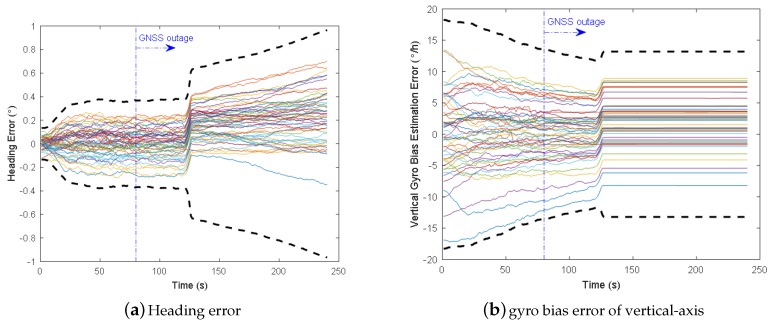
Heading and vertical-axis gyro bias estimation errors for trajectory No.3 when AIME is used (50 trials and 3-sigma error bound).

Monte Carlo simulations are conducted 50 runs for each scheme and each trajectory. Corresponding curved lane detection and heading accuracy results (1-sigma RMS errors at the endpoint) are presented in Table 4. The heading and vertical gyro bias estimation error for the trajectory #3 are illustrated in Figure 17 without AIME and in Figure 18 when AIME is used.

For the straight line detection algorithm (the threshold of second-degree term $a_{0}=1\times 10^{-4}$ in Figure 3 in our case), we find that it can report the curved lane successfully when the curve radius is less than 3150 m (for example the case radius R = 3000 m). When the radius is larger (cases #2 to #4 in Table 4), the straight line detection algorithm will wrongly detect the lane as straight. As a result, VP-aiding demonstrates very large heading and vertical-gyro bias estimation errors. More curved the lane is, larger errors will be. The heading error growth rate is approximately the turning rate in such curved lane. For example, for R = 4584 m, the turning rate is 10 m/s/4584 m = 450${}^{\circ }$/h, and heading error growth rate is $14.18^{\circ }$/120 s = 425.4${}^{\circ }$/h.

When we add the AIME soft fault detection function (AIME detection sample size N is 50 (5 s measurements)), the heading and vertical-gyro bias errors drop significantly compared with VP without AIME. The heading error is slightly larger than the scheme without VP, because VP still works in the 5 s detection period. The fault measurements have the direct influence to heading angle, while the vertical-gyro bias estimation is less influenced by the short fault interval, so we can observe that the vertical-gyro bias error is smaller than the scheme without VP in Table 4. As a result, the AIME is suggested for the soft failure detection in proposed VP-aided INS.

### 5.2. A Complement to Point-Based VO-Aiding

We emphasis that the proposed method is not necessarily superior to the conventional point-based VO. It acts as a complementary method to aid the INS. One of the main advantages of VP-aiding method is that it does not need to track or match the features among frames. Compared to point features, lane markings are "higher level" features containing road direction information. Therefore, our method has the potential to incorporate the digital map to obtain the absolute heading information for additional aiding, which is somehow related with our previous work [29]. However, the disadvantages are also obvious. VP-aiding can only work in the environment with parallel lines, and using the lane VPs only provides one dimension attitude information. For the conventional VO aiding, there is no shape requirement in the scene, and it can output 3-dimentional relative rotation and relative translation (up to scale for monocular case).

The main challenge for conventional monocular VO is the data degeneracy in the pose estimation. The essential (E) matrix is unstable when the feature points lie close to planes, for example, on the ground plane, or when there is no motion or pure rotation of the camera. On the contrary, homography (H) computation requires the feature points on a plane or pure rotation of the camera. To handle this, it is required to detect the degenerate configurations and automatically switch between E and H, which will lead to additional computation cost [16].

As VP-aiding and VO-aiding utilize different environmental features, they can work together to improve accuracy and robustness of the vision-aided INS.

## 6. Conclusions

In this work, we have investigated an alternative approach to mitigate the navigation error growth by using the vanishing point observations to aid the INS for land vehicle navigation applications. The VP module, extracting information from parallel lane markings, is added to the modified NHC/odometer aiding module to further constrain the navigation errors during GNSS outages. The main contributions are the development of VP-aided INS measurement model and the motion constraint aiding module considering the body-to-vehicle frame misalignment. The Monte Carlo simulations and real experiments have shown the smaller heading and position drifting by fusing VP measurements and motion constraint to the navigation system, because of the complementary information provided by these two aiding modules for the INS.

## Figures and Tables

**Table 1:** Sensor Parameters.

Sensors	Items	Values
Gyroscopes (200 Hz)	Bias	36${}^{\circ }$/h (1 σ)
	ARW	$0.6^{\circ }/\sqrt{h}\left(1\;\sigma \right)$
Accelerometers (200 Hz)	Bias	1 mg (1σ)
	VRW	0.05 m/s/$\sqrt{h}\left(1\;\sigma \right)$
Camera (10 Hz)	IOPs	Same with the experiment
	VP accuracy	2 pixels (1 σ)
Speed indicator (odometer)	Scale factor error	0.001 (1 σ)
	Noise standard deviation	0.005 m/s (1 σ)
Relative Pose	IMU/Vehicle misalignment	*x*-axis: $0.8^{\circ }$, *z*-axis: $1^{\circ }$ (1 σ)
	IMU/Vehicle lever-arm	0.1 m in three directions (1 σ)
	camera/vehicle boresight error	$1^{\circ }$ for pitch (1 σ)
GNSS (1 Hz, valid in first 80 s)	Position accuracy	2 m (1 σ)
	Velocity accuracy	0.5 m/s (1 σ)

**Table 2:** Navigation Errors During GPS Outage (path-0028).

	Free INS			INS/NHC			INS/NHC/VP		
	Mean	Max	RMS	Mean	Max	RMS	Mean	Max	RMS
East position (m)	0.918	4.337	1.731	−0.893	2.097	1.086	0.701	1.582	0.840
North position (m)	−4.337	13.23	5.670	−1.327	3.313	1.616	−0.839	2.139	1.026
Height (m)	0.063	0.558	0.282	0.091	0.566	0.267	0.072	0.547	0.271
Pitch (${}^{\circ }$)	0.096	0.216	0.108	0.012	0.103	0.038	0.009	0.103	0.038
Roll (${}^{\circ }$)	−0.032	0.114	0.046	−0.024	0.099	0.045	−0.020	0.095	0.043
Heading (${}^{\circ }$)	−0.241	0.409	0.254	−0.248	0.423	0.262	−0.189	0.423	0.206
East velocity (m/s)	0.089	0.221	0.124	−0.044	0.132	0.069	−0.031	0.122	0.059
North velocity (m/s)	0.315	0.824	0.372	−0.080	0.171	0.091	−0.055	0.132	0.072
Up velocity (m/s)	0.004	0.038	0.022	0.004	0.040	0.021	0.004	0.039	0.021

**Table 3:** Navigation Errors During GPS Outage (path-0101).

	Free INS			INS/NHC			INS/NHC/VP		
	Mean	Max	RMS	Mean	Max	RMS	Mean	Max	RMS
East position (m)	2.768	7.076	3.561	−1.970	4.336	2.355	−1.877	4.1224	2.241
North position (m)	−4.337	13.23	5.670	−1.327	3.313	1.616	−0.839	2.139	1.026
Height (m)	0.063	0.558	0.282	0.091	0.566	0.267	0.072	0.547	0.271
Pitch (${}^{\circ }$)	0.096	0.216	0.108	0.012	0.103	0.038	0.009	0.103	0.038
Roll (${}^{\circ }$)	−0.032	0.114	0.046	−0.024	0.099	0.045	−0.020	0.095	0.043
Heading (${}^{\circ }$)	−0.241	0.409	0.254	−0.248	0.423	0.262	−0.189	0.423	0.206
East velocity (m/s)	0.089	0.221	0.124	−0.044	0.132	0.069	−0.031	0.122	0.059
North velocity (m/s)	0.315	0.824	0.372	−0.080	0.171	0.091	−0.055	0.132	0.072
Up velocity (m/s)	0.004	0.038	0.022	0.004	0.040	0.021	0.004	0.039	0.021

**Table 4:** Simulation Results of Different VP-aiding Schemes under Different Curve Radii (RMS Error).

Trajectory No.	Curve Detected	No VP		With VP		VP & AIME	
		Heading	*z*-Gyro Bias	Heading	*z*-Gyro Bias	Heading	*z*-Gyro Bias
# 1: R = 3000 m	Success	0.27${}^{\circ }$	4.35${}^{\circ }$/h	0.25${}^{\circ }$	3.98${}^{\circ }$/h	–	–
# 2: R = 4584 m	Failed	0.27${}^{\circ }$	4.67${}^{\circ }$/h	14.18${}^{\circ }$	128.99${}^{\circ }$/h	0.34${}^{\circ }$	4.20${}^{\circ }$/h
# 3: R = 6000 m	Failed	0.32${}^{\circ }$	5.45${}^{\circ }$/h	10.87${}^{\circ }$	97.92${}^{\circ }$/h	0.32${}^{\circ }$	4.40${}^{\circ }$/h
# 4: R = 8000 m	Failed	0.28${}^{\circ }$	4.72${}^{\circ }$/h	8.12${}^{\circ }$	73.54${}^{\circ }$/h	0.31${}^{\circ }$	3.99${}^{\circ }$/h

## References

[1] Eckhoff D., Sofra N., German R. A performance study of cooperative awareness in ETSI ITS G5 and IEEE WAVE. Proceedings of the 2013 10th Annual Conference on Wireless On-Demand Network Systems and Services, WONS 2013.

[2] European Telecommunications Standards Institute (2014). Intelligent Transport Systems (ITS)—Vehicular Communications—Basic Set of Applications—Part 2: Specification of Cooperative Awareness Basic Service ETSI EN 302 637-2 V1.3.2.

[3] Petit J., Shladover S.E. (2015). Potential Cyberattacks on Automated Vehicles. IEEE Trans. Intell. Transp. Syst..

[4] Liu Y., Fu Q., Liu Z., Li S. GNSS spoofing detection ability of a loosely coupled INS/GNSS integrated navigation system for two integrity monitoring methods. Proceedings of the 2017 International Technical Meeting of the Institute of Navigation, ITM 2017.

[5] Dissanayake G., Sukkarieh S., Nebot E., Durrant-Whyte H. (2001). The aiding of a low-cost strapdown inertial measurement unit using vehicle model constraints for land vehicle applications. IEEE Trans. Robot. Autom..

[6] Niu X., Nassar S., El-Sheimy N. (2007). An Accurate Land-Vehicle MEMS IMU/GPS Navigation System Using 3D Auxiliary Velocity Updates. J. Inst. Navig..

[7] Atia M.M., Liu S., Nematallah H., Karamat T.B., Noureldin A. (2015). Integrated indoor navigation system for ground vehicles with automatic 3-D alignment and position initialization. IEEE Trans. Veh. Technol..

[8] Yu C., El-Sheimy N., Lan H., Liu Z. (2017). Map-Based Indoor Pedestrian Navigation Using an Auxiliary Particle Filter. Micromachines.

[9] Attia M., Moussa A., El-Sheimy N. Bridging integrated GPS/INS systems with geospatial models for car navigation applications. Proceedings of the 23rd International Technical Meeting of the Satellite Division of the Institute of Navigation (ION GNSS 2010).

[10] Tardif J.P., George M., Laverne M., Kelly A., Stentz A. A new approach to vision-aided inertial navigation. Proceedings of the 2010 IEEE/RSJ International Conference on Intelligent Robots and Systems (IROS).

[11] Schmid K., Ruess F., Suppa M., Burschka D. State estimation for highly dynamic flying systems using key frame odometry with varying time delays. Proceedings of the IEEE International Conference on Intelligent Robots and Systems.

[12] Veth M.J. (2008). Fusion of Imaging and Inertial Sensors for Navigation. Ph.D. Thesis.

[13] Mourikis A.I., Roumeliotis S.I. (2007). A multi-state constraint Kalman filter for vision-aided inertial navigation. Proceedings of the IEEE International Conference on Robotics and Automation.

[14] Leutenegger S., Lynen S., Bosse M., Siegwart R., Furgale P. (2015). Keyframe-based visual-inertial odometry using nonlinear optimization. Int. J. Robot. Res..

[15] Caprile B., Torre V. (1990). Using vanishing points for camera calibration. Int. J. Comput. Vis..

[16] Bazin J.C., Demonceaux C., Vasseur P., Kweon I.S. (2010). Motion estimation by decoupling rotation and translation in catadioptric vision. Comput. Vis. Image Underst..

[17] Keßler C., Ascher C., Flad M., Trommer G.F. (2012). Multi-sensor indoor pedestrian navigation system with vision aiding. Gyroscopy Navig..

[18] Ruotsalainen L., Kuusniemi H., Bhuiyan M.Z.H., Chen L., Chen R. (2013). A two-dimensional pedestrian navigation solution aided with a visual gyroscope and a visual odometer. GPS Solut..

[19] Camposeco F., Pollefeys M. Using vanishing points to improve visual-inertial odometry. Proceedings of the 2015 IEEE International Conference on Robotics and Automation (ICRA).

[20] Williams B., Hudson N., Tweddle B., Brockers R., Matthies L. Feature and pose constrained visual Aided Inertial Navigation for computationally constrained aerial vehicles. Proceedings of the 2011 IEEE International Conference on Robotics and Automation.

[21] Hwangbo M., Kanade T. Visual-inertial UAV attitude estimation using urban scene regularities. Proceedings of the IEEE International Conference on Robotics and Automation.

[22] Kim S.B., Bazin J.C., Lee H.K., Choi K.H., Park S.Y. (2011). Ground vehicle navigation in harsh urban conditions by integrating inertial navigation system, global positioning system, odometer and vision data. IET Radar Sonar Navig..

[23] Bazin J.C., Demonceaux C., Vasseur P., Kweon I. (2012). Rotation estimation and vanishing point extraction by omnidirectional vision in urban environment. Int. J. Robot. Res..

[24] Schwarze T., Lauer M. Robust ground plane tracking in cluttered environments from egocentric stereo vision. Proceedings of the IEEE International Conference on Robotics and Automation.

[25] Lee B., Zhou J., Ye M., Guo Y., Sensing R., Calibration C., Phone S. A Novel Approach to Camera Calibration Method for Smart Phones. Proceedings of the the International Archives of the Photogrammetry, Remote Sensing and Spatial Information Sciences.

[26] Seo Y.W., Rajkumar R.R. Use of a Monocular Camera to Analyze a Ground Vehicle’s Lateral Movements for Reliable Autonomous City Driving. Proceedings of the IEEE IROS Workshop on Planning, Perception and Navigation for Intelligent Vehicles.

[27] Tao Z., Bonnifait P., Fremont V., Ibanez-Guzman J. Lane marking aided vehicle localization. Proceedings of the IEEE Conference on Intelligent Transportation Systems, ITSC.

[28] Cui D., Xue J., Zheng N. (2016). Real-Time Global Localization of Robotic Cars in Lane Level via Lane Marking Detection and Shape Registration. IEEE Trans. Intell. Transp. Syst..

[29] Liu Z., El-sheimy N., Yu C., Qin Y. Vanishing Point/Vehicle Motion Constraints Aided Ground Vehicle Navigation. Proceedings of the 2017 International Technical Meeting of the Institute of Navigation.

[30] Chatzi E.N., Smyth A.W. (2009). The unscented Kalman filter and particle filter methods for nonlinear structural system identification with non-collocated heterogeneous sensing. Struct. Control Health Monit..

[31] Wendel J., Metzger J., Moenikes R., Maier A., Trommer G.F. (2006). A Performance comparison of tightly coupled GPS/INS navigation systems based on extended and sigma point Kalman filters. Navig. J. Inst. Navig..

[32] Shin E.H., El-Sheimy N. (2007). Unscented Kalman Filter and Attitude Errors of Low-Cost Inertial Navigation Systems. Navigation.

[33] Eftekhar Azam S., Chatzi E., Papadimitriou C. (2015). A dual Kalman filter approach for state estimation via output-only acceleration measurements. Mech. Syst. Signal Process..

[34] Azam S.E., Chatzi E., Papadimitriou C., Smyth A. (2017). Experimental validation of the Kalman-type filters for online and real-time state and input estimation. J. Vib. Control.

[35] Roumeliotis S.I., Burdick J.W. Stochastic cloning: A generalized framework for processing relative state measurements. Proceedings of the 2002 IEEE International Conference on Robotics and Automation.

[36] Mourikis A.I., Roumeliotis S.I., Burdick J.W. (2007). SC-KF Mobile Robot Localization : A Stochastic-Cloning Kalman Filter for Processing Relative-State Measurements. IEEE Trans. Robot..

[37] Popp M., Crocoll P., Ruppelt J., Trommer G.F. A Novel Multi Image Based Navigation System to Aid Outdoor—Indoor Transition Flights of Micro Aerial Vehicles. Proceedings of the 27th International Technical Meeting of the Satellite Division of the Institute of Navigation ION GNSS+.

[38] Liu Z., El-Sheimy N., Qin Y. Low-cost INS/Odometer Integration and Sensor-to-sensor Calibration for Land Vehicle Applications. Proceedings of the IAG/CPGPS International Conference on GNSS+ (ICG+ 2016).

[39] Hartley R., Zisserman A. (2003). Multiple View Geometry in Computer Vision.

[40] Bar Hillel A., Lerner R., Levi D., Raz G. (2014). Recent progress in road and lane detection: A survey. Mach. Vis. Appl..

[41] El-Sheimy N. (2016). Inertial Surveying and INS/GPS Integration, Lecture Notes for ENGO 623 Course.

[42] Qin Y. (2014). Inertial Navigation.

[43] Liu Z., El-Sheimy N., Qin Y., Yu C., Zhang J., Sun J., Liu J., Fan S., Wang F. (2016). Partial State Feedback Correction for Smoothing Navigational Parameters. China Satellite Navigation Conference (CSNC) 2016 Proceedings: Volume II, Changsha, China, 18–20 May 2016.

[44] Liu Z., Qin Y., Li S., Cui X. A new IMU-based method for relative pose determination. Proceedings of the 22nd Saint Petersburg International Conference on Integrated Navigation Systems.

[45] Geiger A., Lenz P., Stiller C., Urtasun R. (2013). Vision meets robotics: The KITTI dataset. Int. J. Robot. Res..

[46] OXTS RT3000 Brochure. https://www.oxts.com/app/uploads/2017/07/RT3000-brochure-170606.pdf.

[47] Groves P.D. (2008). Principles of GNSS, Inertial, and Multisensor Integrated Navigation Systems.

